# The nucleocapsid proteins of mouse hepatitis virus and severe acute respiratory syndrome coronavirus share the same IFN-β antagonizing mechanism: attenuation of PACT-mediated RIG-I/MDA5 activation

**DOI:** 10.18632/oncotarget.17912

**Published:** 2017-05-17

**Authors:** Zhen Ding, Liurong Fang, Shuangling Yuan, Ling Zhao, Xunlei Wang, Siwen Long, Mohan Wang, Dang Wang, Mohamed Frahat Foda, Shaobo Xiao

**Affiliations:** ^1^ State Key Laboratory of Agricultural Microbiology, College of Veterinary Medicine, Huazhong Agricultural University, Wuhan 430070, China; ^2^ Key Laboratory of Preventive Veterinary Medicine in Hubei Province, The Cooperative Innovation Center for Sustainable Pig Production, Wuhan 430070, China

**Keywords:** coronavirus, nucleocapsid protein, innate immunity evasion, type I interferon, PACT

## Abstract

Coronaviruses (CoVs) are a huge threat to both humans and animals and have evolved elaborate mechanisms to antagonize interferons (IFNs). Nucleocapsid (N) protein is the most abundant viral protein in CoV-infected cells, and has been identified as an innate immunity antagonist in several CoVs, including mouse hepatitis virus (MHV) and severe acute respiratory syndrome (SARS)-CoV. However, the underlying molecular mechanism(s) remain unclear. In this study, we found that MHV N protein inhibited Sendai virus and poly(I:C)-induced IFN-β production by targeting a molecule upstream of retinoic acid-induced gene I (RIG-I) and melanoma differentiation gene 5 (MDA5). Further studies showed that both MHV and SARS-CoV N proteins directly interacted with protein activator of protein kinase R (PACT), a cellular dsRNA-binding protein that can bind to RIG-I and MDA5 to activate IFN production. The N–PACT interaction sequestered the association of PACT and RIG-I/MDA5, which in turn inhibited IFN-β production. However, the N proteins from porcine epidemic diarrhea virus (PEDV) and porcine reproductive and respiratory syndrome virus (PRRSV), which are also classified in the order *Nidovirales*, did not interact and counteract with PACT. Taken together, our present study confirms that both MHV and SARS-CoV N proteins can perturb the function of cellular PACT to circumvent the innate antiviral response. However, this strategy does not appear to be used by all CoVs N proteins.

## INTRODUCTION

Coronaviruses (CoVs) are a type species of enveloped viruses with a single-stranded positive RNA genome of approximately 26–32 kilobases. CoVs belong to the *Coronaviridae* family, classified into the order *Nidovirales* along with *Arteriviridae* [1, 2]. All CoVs share similarities in the organization and expression of their genome, which is arranged into open reading frame (ORF) 1a, ORF1b, S, ORF3, E, M and N in order, with both termini flanked by 5′- and 3′-untranslated regions. CoVs are separated into four genera based on phylogeny: *alpha-CoV*, *beta-CoV*, *gamma-CoV* and *delta-CoV*. The beta-CoV genus contains important veterinary and human etiological agents, such as mouse hepatitis virus (MHV) and severe acute respiratory syndrome coronavirus (SARS-CoV). MHV infects mice liver or neuron, but it is a well-studied coronavirus model for similar patterns of pathogenesis [5]. SARS-CoV is the etiological agent behind an outbreak of severe respiratory disease through China during 2002–2003 [6–8].

Virus infection can be sensed in host cells through the interaction of pathogen-associated molecular patterns with host pattern recognition receptors [13]. During CoVs infection, viral components or replication intermediates can be detected by retinoic acid-induced gene I (RIG-I) and melanoma differentiation gene 5 (MDA5) [14–17]. Activated RIG-I and/or MDA5 recruit the mitochondrial adaptor protein MAVS/IPS-1/Cardif through caspase activation and recruitment domain (CARD)–CARD interactions to activate the downstream κB kinase (IKK)-related kinases, such as TANK-binding kinase 1 (TBK1) and IKKε. The TBK1-IKKε complex then mediates downstream activation of the critical transcription factors, interferon regulation factor 3 (IRF3) and nuclear factor κB (NF-κB), triggering their nuclear translocation, where they form active transcriptional complexes that bind to IFN promoter elements and activate type I IFN gene expression [18–20]. Recent studies have identified a new molecule that acts as a protein activator of protein kinase R (PKR), namely protein activator of the interferon-induced protein kinase (PACT) [21, 22], and also promotes RIG-I signaling in response to viral infection and dsRNA through interacting with the carboxy-terminal domain (CTD) of RIG-I, which in turn stimulates RIG-I ATPase activity [23, 24].

Many CoVs, including MHV, SARS-CoV, Middle East respiratory symdrome (MERS)-CoV, porcine epidemic diarrhea virus (PEDV), and porcine deltacoronavirus (PDCoV), have been reported to cause dysregulated type I IFN expression to counteract the host innate immune defense, and this is mostly due to virus produced-proteins involved in IFN evasion [25–35]. Nucleocapsid (N) proteins of CoVs are a substantial part of the viral proteins, seemingly dedicated to suppressing the antiviral response [36]. SARS-CoV and PEDV N proteins can inhibit IFN-β synthesis, and this occurs by an unknown mechanism in SARS-CoV [37]. We have previously found that PEDV N protein blocks IFN-β signaling by targeting TBK1 to prevent its interaction with IRF3. We also investigated whether MHV and SARS-CoV N proteins use the same strategy to antagonize IFN-β production; however, the N proteins of MHV and SARS-CoV do not interact with TBK1 [38].

In the present study, we showed that MHV N protein is responsible for IFN-β inhibition, and exerts its IFN-antagonizing function by targeting PACT, thereby impairing PACT-RIG-I/MDA5 interactions and RIG-I/MDA5 activation for downstream IFN production. This is the same evasion strategy developed by SARS-CoV N protein. However, this mechanism was not shared by PEDV or porcine reproductive and respiratory syndrome virus (PRRSV) N proteins. Our findings thus shed light on the IFN-regulatory mechanism employed by beta-CoV N proteins.

## RESULTS

### MHV N protein blocks Sendai virus-induced IFN-β production

**Figure 1:**
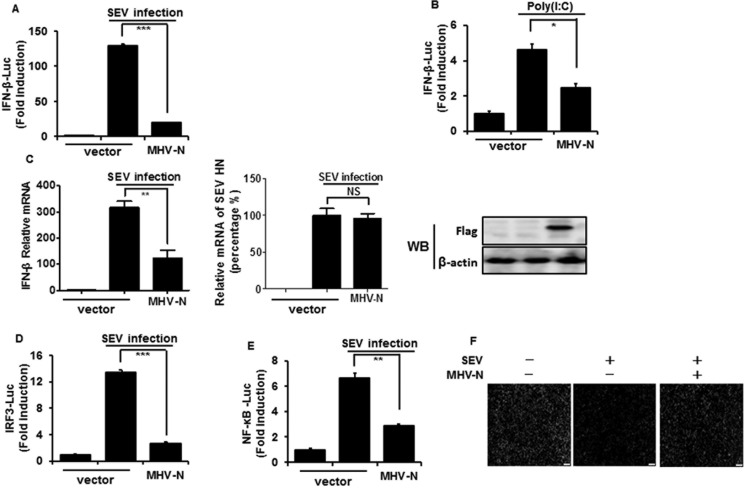
MHV N protein inhibits SEV-induced IFN-β production HEK-293T cells were co-transfected with IFN-β–Luc (**A** and **B**), IRF3–Luc (**D**), or NF-κB–Luc (**E**), together with the pRL–TK plasmid and MHV-N expression vector. Twenty-four hours after the initial transfection, cells were infected with SEV (A, D, E) or treated with poly(I:C) (B). Luciferase assays were performed 12 h after infection or treatment. The results represent the means and standard deviations of three independent experiments. The relative firefly luciferase activity was normalized to the *Renilla reniformis* luciferase activity, and the uninfected or untreated empty vector control value was set to 1. (**C**) HEK-293T cells were transfected with MHV-N expression plasmid. After infection with SEV for 12 h, cells were lysed to extract total RNA, which was used for detecting the expression of the IFN-β, SEV HN and GAPDH genes by quantitative real-time RT-PCR. The results are expressed as increases in mRNA levels or SEV HN gene copies relative to those in cells transfected without SEV infection and were normalized to the expression of the GAPDH housekeeping gene. (**F**) HEK-293T cells were transfected with MHV-N expression plasmid and then infected with SEV for 12 h. The cells were then re-infected with VSV-GFP for 24 h, followed by analysis for fluorescence by microscopy. *P < 0.05; **P < 0.01; ***P < 0.001.

The MHV N protein has been reported to block type I IFN-induced antiviral responses, but the effect on IFN-β production is still unclear [39]. To test this, HEK-293T cells were co-transfected with a DNA expression construct pCMV-tag2b-MHV-N encoding MHV N protein and luciferase reporter plasmid IFN-β–Luc, together with the internal control plasmid pRL–TK, and then infected with Sendai virus (SEV) or treated with poly(I:C). As shown in Figure 1A and 1B, the overexpression of MHV N protein blocked the SEV/poly(I:C)-induced IFN-β promoter activities. To further confirm that IFN-β expression was inhibited by MHV N protein, HEK-293T cells were transiently transfected with pCMV-tag2b-MHV-N plasmid and then infected with SEV for 12 h. Cells were collected for detection of IFN-β relative mRNA expression and SEV NH gene copies by real-time RT-PCR. The expression of MHV N protein was detected by immunoblotting with an anti-Flag antibody. As shown in Figure 1C, SEV-induced mRNA expression of IFN-β was significantly reduced in MHV N protein-expressing cells, meanwhile SEV NH RNA in mock cells equaled to cells expressing MHV N protein. This indicated MHV N perturbed IFN-β production not result from inhibiting SEV replication. The coordinated and cooperative actions of transcription factors IRF3 and NF-κB are essential to mediate type I IFN expression [40, 41], therefore IRF3 and NF-κB were additionally examined to investigate the role of MHV N protein in type I IFN suppression. Luciferase reporter gene assays were performed with luciferase reporter plasmids IRF-3–Luc (Figure 1D) or NF-κB–Luc (Figure 1E). Our results indicated that SEV-induced promoter activities of IRF-3 and NF-κB are also blocked by MHV N protein.

SEV-induced IFNs initiate a series of signaling cascades through the Janus kinase/signal transducer and activator of transcription pathway to direct antiviral and immune gene expression, resulting in the suppression of virus replication [42]. Vesicular stomatitis virus encoding green fluorescent protein (VSV-GFP) is sensitive to this antiviral response [43–45], which makes it a good model system to screen IFN-β antagonists. To further confirm the inhibitory effect of MHV N protein on the SEV-induced antiviral response, HEK-293T cells were transfected or mock transfected with pCMV-tag2b-MHV-N plasmid prior to infection with SEV and VSV-GFP. Dramatic reduction of VSV-GFP proliferation was observed after SEV infection, but a robust restorative effect was observed in N protein-expressing cells (Figure 1F). These results further confirmed that MHV N protein antagonizes the production of IFN-β.

### MHV N protein blocks IFN-β production by targeting molecule(s) upstream of RIG-I and MDA5

**Figure 2:**
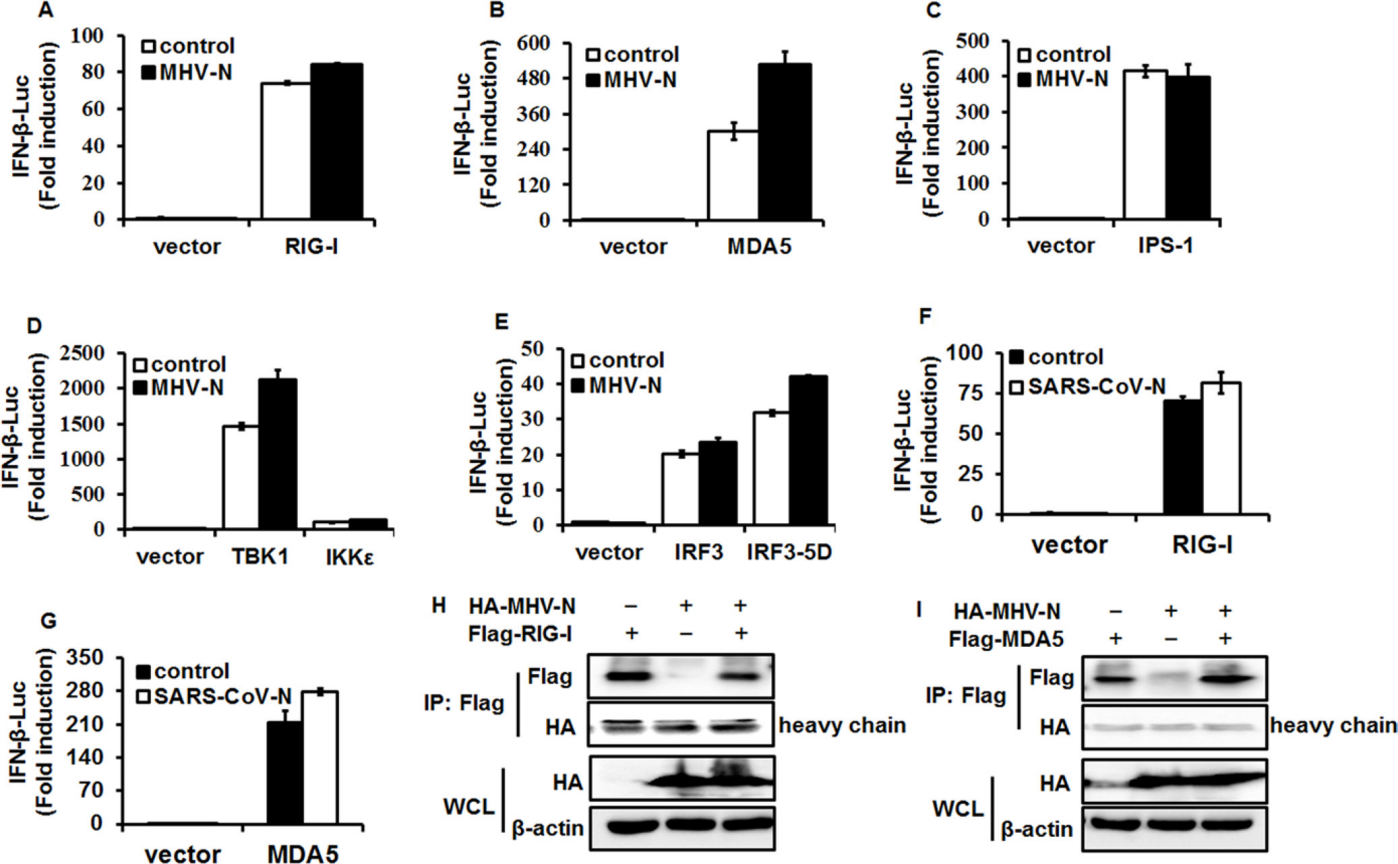
MHV N protein antagonizes IFN-β production by targeting molecule(s) upstream of RIG-I/MDA5 HEK-293T cells were co-transfected with IFN-β–Luc, pRL–TK plasmids, and plasmids encoding MHV-N (**A**–**E**) or SARS-N (**F** and **G**) proteins together with constructs expressing RIG-I, MDA5, IPS-1, IKKe, TBK1, IRF3 and IRF3-5D. Luciferase assays were performed at 28 h after transfection. The results represent the means and standard deviations of three independent experiments. (**H** and **I**). pCAGGS–HA-MHV-N and Flag-RIG-I or -MDA5 vectors were transfected into HEK-293T cells and immunoprecipitation was performed with an anti-Flag antibody, followed by immunoblotting analyses with antibodies against Flag, HA and β-actin, respectively.

RIG-I and MDA5 are RNA sensors and can be activated by their respective RNA ligands to induce the downstream cascade pathway [46, 47]. To identify the molecular target of MHV N protein in the IFN-induction signaling pathway, HEK-293T cells were co-transfected with pCMV-tag2b-MHV-N vector and luciferase reporter plasmids containing the IFN-β promoter and Renilla luciferase-expressing plasmid, pRL–TK, and a series of IFN-β promoter stimulators in the RIG-I-like receptor (RLR) signaling pathway, including RIG/RIG-IN, MDA5, IPS-1, TBK1, IKKε, IRF3 and IRF3-5D. Luciferase assays were performed at 28 h after co-transfection. The overexpression of any molecule in the RLR signaling pathway dramatically stimulated IFN-β promoter activation, but interestingly, MHV N had no inhibitory effect on this activation (Figure 2A–2E). This is coincident with SARS-CoV N protein inhibition of IFN-β production, which also fails to affect the activation of these signaling molecules. We re-confirmed that overexpression of SARS-CoV N protein did not inhibit IFN-β promoter activation induced by RIG-I (Figure 2F) and MDA5 (Figure 2G) under our experimental conditions. If MHV N protein involved the identical IFN-β-antagonizing mechanisms to SARS-CoV N protein, which has been demonstrated not interact with RIG-I or MDA5 [37], MHV N protein should not be supposed to target RIG-I or MDA5. To test this hypothesis, expression plasmids of hemagglutinin (HA)-tagged MHV N and Flag-tagged RIG-I (Figure 2H) or MDA5 (Figure 2I) were co-transfected into HEK-293T cells. At 28 h posttransfection, co-immunoprecipitations were performed with anti-Flag antibodies. As speculated, MHV N protein was not detected in neither Flag-tagged RIG-I nor MDA5 precipitates, suggesting that MHV N protein did not associate with RIG-I or MDA5. All together, these data indicated that MHV N and SARS-CoV N proteins suppress the activation of an upstream molecule in the IFN signaling cascade.

### MHV and SARS-CoV N proteins attenuate PACT-mediated RIG-I/MDA5 activation of the IFN-β promoter

**Figure 3:**
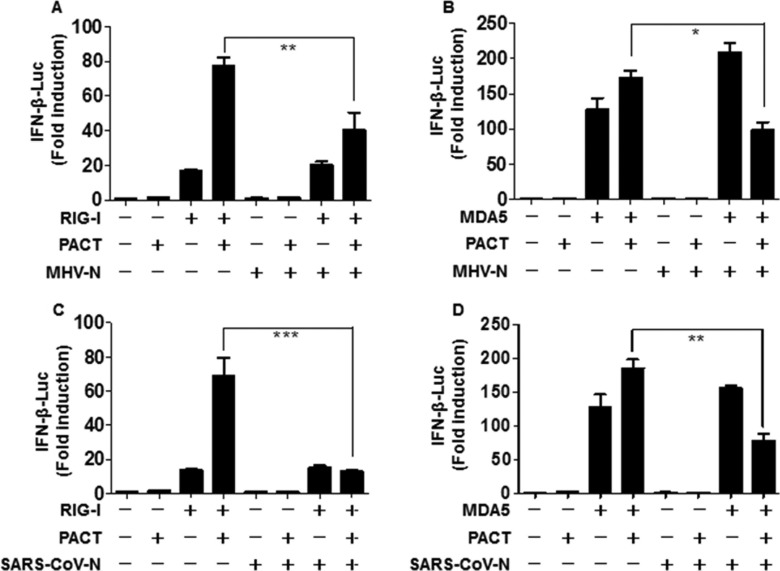
The N proteins of MHV and SARS-CoV inhibit PACT-mediated activation of RIG-I/MDA5 HEK-293T cells grown in 24-well plates were transfected with expression plasmids for RIG-I (**A**, **C**) or MDA5 (**B**, **D**), PACT, and N proteins from MHV (A, B) or SARS-CoV (C, D) as well as IFN-β–Luc and pRL–TK reporter plasmids. The dual-luciferase assays were carried out at 28 h post-transfection. The results represent the means and standard deviations of three independent experiments. *P < 0.05; ***P < 0.001.

Previous studies have revealed that optimal activity of RIG-I and MDA5 requires PACT, a cellular dsRNA-binding protein which binds to RIG-I and MDA5 to induce IFN production [24, 48, 49]. MHV and SARS-CoV N proteins with RIG-I-signaling antagonistic and RNA binding properties were speculated to target PACT. To test this possibility, HEK-293T cells were co-transfected with MHV N or SARS-CoV N expression plasmids and PACT, and either RIG-I or MDA5, along with luciferase reporter plasmids. Luciferase assays were performed to assess the suppressive activity in the presence of PACT and either RIG-I or MDA5 at 28 h after co-transfection. As shown in Figure 3, PACT promoted the activation of RIG-I and MDA5 for induction of IFN-β promoter activity, but this effect was reversed by the N proteins of MHV and SARS-CoV. These findings indicated that MHV (Figure 3A and 3B) and SARS-CoV (Figure 3C and 3D) N proteins counteract the activity of PACT.

### MHV and SARS-CoV N proteins bind to PACT and impair interactions between PACT and RIG-I/MDA5

**Figure 4:**
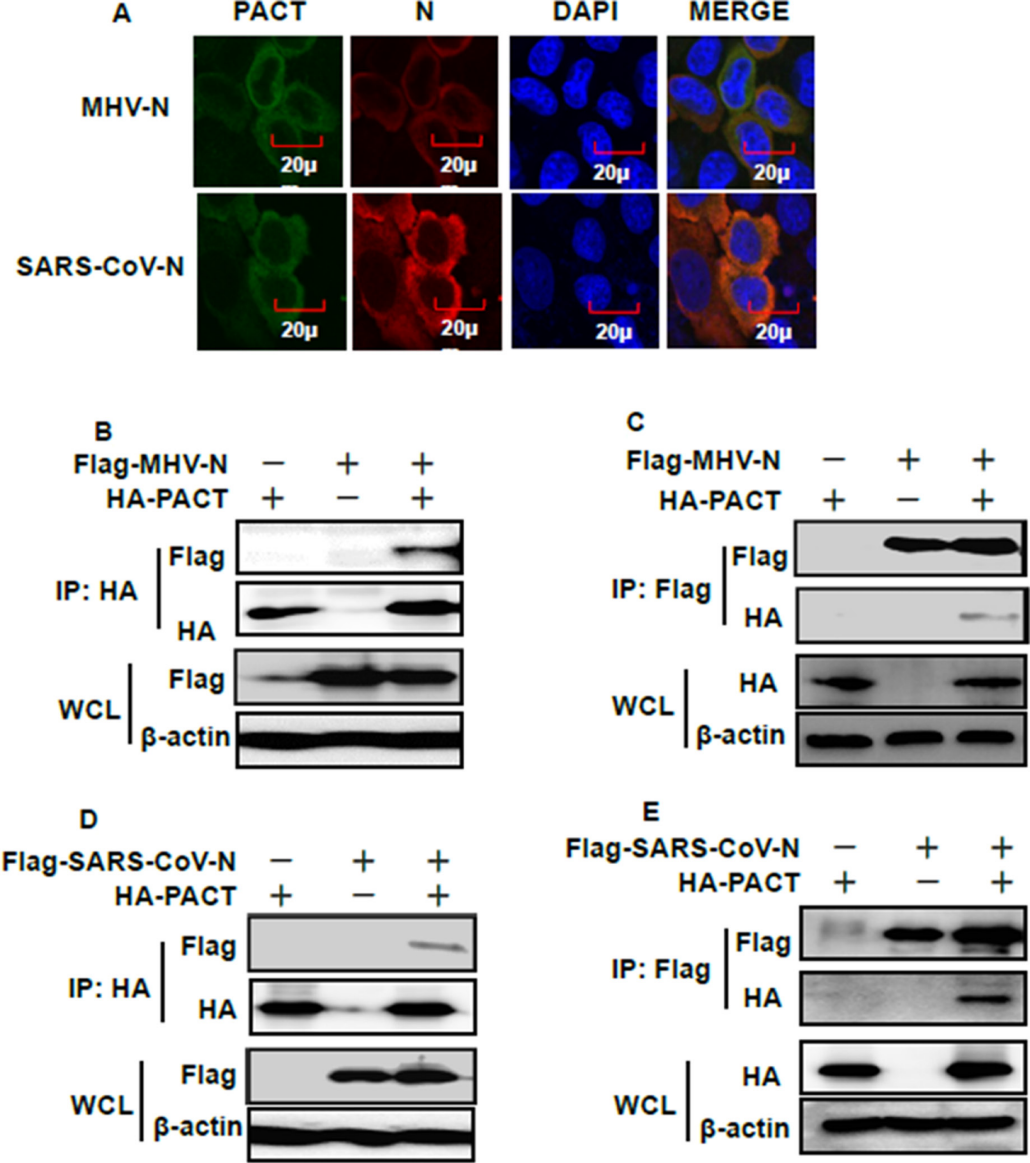
PACT interacts with the N proteins of MHV and SARS-CoV (**A**) HEK-293T cells were transfected with expression plasmids encoding HA-tagged PACT and Flag-tagged MHV or SARS-CoV N proteins. At 28 h after transfection, cells were then fixed for IFA to detect PACT (green) and N proteins (red) with anti-HA and anti-Flag antibodies, respectively. DAPI (blue) indicates the locations of the cell nuclei. Fluorescent images were acquired with a confocal laser scanning microscope (Olympus Fluoview ver. 3.1, Japan). (**B** and **D**) HEK-293T cells were transfected with expression constructs encoding HA-tagged PACT and Flag-tagged MHV or SARS-CoV N proteins. The cells were lysed at 28 h after transfection and subjected to immunoprecipitation with anti-HA antibody. The whole-cell lysates (WCL) and immunoprecipitation (IP) complexes were analyzed by immunoblotting using anti-Flag, anti-HA, or anti-β-actin antibodies. (**C** and **E**) Transient transfection and immunoblotting analyses were carried out in the manner described in B and D above, but immunoprecipitation was performed with anti-Flag antibody.

If MHV or SARS-CoV N proteins target PACT, then colocalization between these proteins should occur within cells. To test this association, Flag-tagged MHV or SARS-CoV N proteins and HA-tagged PACT were co-transfected into HEK-293T cells. Indirect immunofluorescence assay (IFA), reciprocal co-immunoprecipitation and immunoblotting experiments were then performed. IFA showed that MHV and SARS-CoV N proteins were both distributed in the cytoplasm, as was PACT (Figure 4A). Co-immunoprecipitation and immunoblotting experiments found that PACT was present in the MHV and SARS-CoV N protein precipitates and *vice versa*, MHV and SARS-CoV N proteins were present in the PACT precipitate (Figure 4B–4E). These results indicated that MHV and SARS-CoV N proteins can interact with PACT.

**Figure 5:**
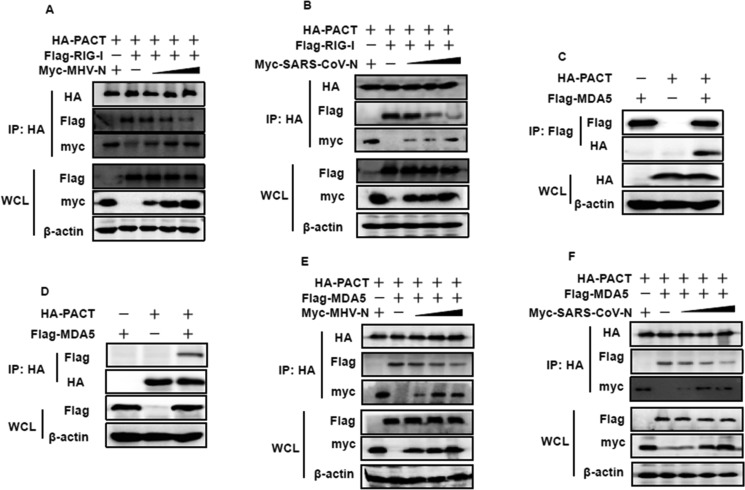
MHV and SARS-CoV N proteins inhibit the interaction between PACT and RIG-I/MDA5 (**A**, **B**, **E** and **F**) HEK-293T cells were co-transfected with HA-tagged PACT and Flag-tagged RIG-I/MDA5 vectors, and increasing amounts (0, 1.5, 3, or 6 μg) of pCAGGS-Myc-MHV-N or pCAGGS-Myc-SARS-CoV-N expressing plasmids. Twenty-eight hours after transfection, the cells were harvested for immunoprecipitation with anti-HA antibodies. WCL and IP complexes were analyzed by immunoblotting (IB) with antibodies against HA, Flag, Myc or β-actin. (**C** and **D**) HEK-293T cells were co-transfected with plasmids expressing HA-tagged PACT and Flag-tagged MDA5. Twenty-eight hours after transfection, immunoprecipitation was carried out with anti-HA or anti-Flag antibodies. WCL and precipitates were probed by immunoblotting analyses with anti-Flag, anti-HA and β-actin antibodies.

PACT physically binds to the C-terminal repression domain of RIG-I and potently stimulates RIG-I-induced type I interferon production, resulting in stimulation of its ATPase activity [24]. N protein–PACT interactions may impair the association between PACT and RIG-I. To assess this hypothesis, HEK-293T cells were transfected with increasing amounts of Myc-tagged MHV or SARS-CoV N proteins, together with constant amounts of pCAGGS–HA-PACT and Flag-tagged RIG-I. Immunoprecipitation was performed with anti-HA antibody, and the co-precipitated N proteins and RIG-I were analyzed by immunoblotting with anti-Myc and anti-Flag antibodies. As shown in Figure 5A and 5B, PACT efficiently pulled down RIG-I. However, the amount of RIG-I present in PACT precipitates gradually decreased as the amount of MHV and SARS-CoV N proteins increased. These results suggested that MHV and SARS-CoV N proteins inhibit the PACT–RIG-I interaction in a concentration-dependent manner.

Considering that co-expression of MDA5 and PACT resulted in enhancement of MDA5-induced activation of the IFN-β promoter [48, 49], we hypothesized that the activation of MDA5 might plausibly be mediated by a direct interaction with PACT. We therefore performed co-immunoprecipitation experiments with lysates of co-transfected or single vector HA-tagged PACT and Flag-tagged MDA5 transfected HEK-293T cells. As shown in Figure 5C and 5D, both HA-tagged PACT and Flag-tagged MDA5 proteins were found to co-precipitate upon detection with anti-HA and anti-Flag antibodies. This indicated that PACT also interact with MDA5. To investigate this interaction further, we repeated the co-immunoprecipitation experiment to detect whether N-PACT interactions could also disrupt the PACT-MDA5 association. Likewise, gradually decreasing levels of MDA5 were detected in the anti-HA precipitate as MHV and SARS-CoV N protein concentrations were increased (Figure 5E and 5F), indicating that formation of the PACT–MDA5 complex is impaired by the viral N proteins.

### MHV and SARS-CoV N proteins interactions with PACT are not completely RNA-dependent

**Figure 6:**
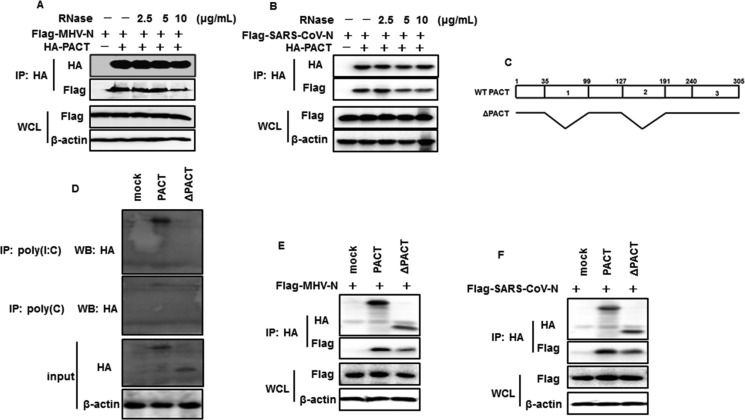
RNase treatment attenuates interactions between PACT and N proteins of MHV and SARS-CoV (**A** and **B**) HEK-293T cells were co-transfected with HA-tagged PACT and Flag-tagged MHV-N or SARS-CoV-N. Immunoprecipitation was performed with anti-HA antibody. Precipitates incubated with increasing amounts of RNase (2.5, 5, 10 μg/mL) in 37°C for 1 hour before washed. (**C**) Architectures of wild type PACT and deletion constructs. WT PACT and ΔPACT refer to wild type PACT and doman1 and domain 2 deleted mutant. Numbers up the box mean PACT amino acid residues. 1, 2, and 3 in the bar indicate PACT domain 1, domain 2 and domain 3, respectively. (**D**) HEK-293T cells were transfected with HA-tagged PACT or ΔPACT expression plasmids for 28 h. Cell lysates were immunoprecipitated with poly(I:C)- or poly(C)-coated agarose in 4°C or 4 h. lysates and precipitates were subject to immunoblotting analysis using anti-HA and β-actin antibodies. (**E** and **F**) HEK-293T cells were co-transfected with plasmids expressing HA-tagged PACT or ΔPACT and Flag-tagged MHV or SARS-CoV N plasmids, followed by immunoprecipitation and immunoblot with anti-HA or/and anti-Flag and β-actin antibodies.

Since MHV and SARS-CoV N proteins and PACT are dsRNA-binding proteins [50], we questioned whether the interactions between N proteins and PACT were mediated by RNA. The co-immunoprecipitation experiments were repeated in the presence of increasing amount of RNase A. The amounts of MHV and SARS-CoV N proteins detected in the PACT precipitates were gradually reduced but not vanished with increasing dose of RNase A (Figure 6A and 6B), indicating that MHV or SARS-CoV N protein can directly RNA-independently interact with PACT. However, N-PACT interaction was weakened when endogenous RNA was digested. PACT contains three domains (domain 1 to 3). Domain 1 and domain 2 possess dsRNA binding activity, while domain 3 is not capable of binding RNA [51]. Theoretically, PACT mutant with RNA binding domain deletion could also interact with MHV and SARS-CoV N proteins. To test this, PACT mutant (ΔPACT) deleting domain 1 and domain 2 was constructed (Figure 6C) and verified no RNA binding activity (Figure 6D). Then ΔPACT-N association was assessed with co-immunoprecipitation experiment using anti-HA antibodies. As shown in Figure 6E and 6F, both MHV and SARS-CoV N proteins were detected in ΔPACT precipitates, with a weaker band compared with wild type PACT precipitates. This confirmed that PACT mutant with no RNA binding activity is sufficient for N interaction.

### PEDV and PRRSV N proteins do not counteract PACT

**Figure 7:**
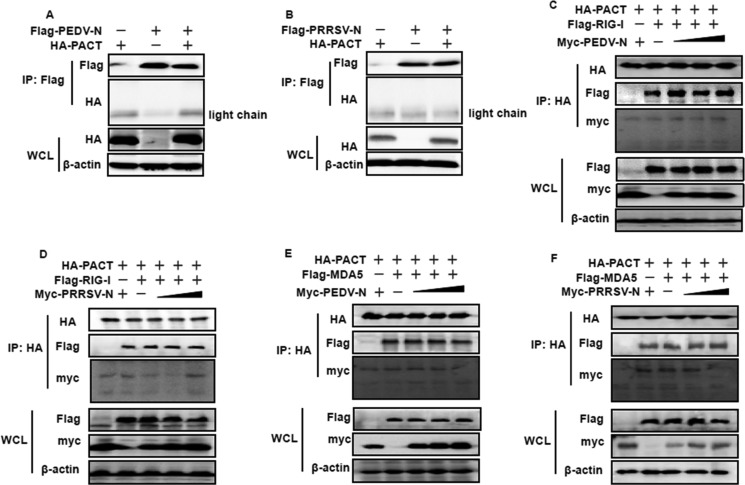
PEDV and PRRSV N proteins do not counteract PACT (**A** and **B**) HEK-293T cells were co-transfected with HA-tagged PACT and Flag-tagged PEDV-N or PRRSV-N. Cell lysates were subjected to immunoprecipitation and immunoblot with anti-Flag or/and anti-HA and β-actin antibodies. (**C**–**F**) HEK-293T cells were co-transfected with HA-tagged PACT and Flag-tagged RIG-I/MDA5 vectors, and increasing amounts (0, 1.5, 3, or 6 μg) of pCAGGS-Myc-PEDV-N or pCAGGS-Myc-PRRSV-N expressing plasmids. Twenty-eight hours after transfection, the cells were harvested for immunoprecipitation with anti-HA antibodies. WCL and IP complexes were analyzed by immunoblotting (IB) with antibodies against HA, Flag, Myc or β-actin.

We wished to determine whether this N–PACT targeting mechanism was universal for CoV-encoded N proteins, and potentially even commonly used by the N proteins from arteriviruses, which are of the same phylogenetic order as CoVs. To test this possibility, the N proteins of PEDV, a member of the alpha-CoV genus and PRRSV, a member of the *arteriviridae* family, were chosen for co-immunoprecipitation experiments; both N proteins are also known to be IFN-antagonizing proteins but function downstream of RIG-I and MDA5 [38, 52]. As shown in Figure 7A and 7B, neither PEDV nor PRRSV N protein was present in the PACT precipitates. Thus, PEDV and PRRSV N proteins were not supposed to impair PACT-RIG-I/MDA5 complex conformation. To test this, Flag-tagged RIG-I or MDA5 expression plasmids, HA-tagged PACT, together with increasing amounts of pCAGGS-Myc-PEDV-N or pCAGGS-Myc-PRRSV-N vectors, were co-transfected into HEK-293T cells for 28 h. Cell lysates were harvested for co-immunoprecipitation experiments using anti-HA antibodies. As shown in Figure 7C–7F, neither PEDV nor PRRSV N proteins disrupted RIG-I or MDA5 solid interaction with PACT. Hence, these supported the notion that MHV and SARS-CoV N proteins are unique in their counteraction with PACT.

## DISCUSSION

Previously, MHV N protein was reported to antagonize the IFN response, with limited information about MHV N protein effects on IFN synthesis. In this study, we found that MHV N protein was responsible for this IFN-antagonizing activity and the molecular mechanism was identical to that of SARS-CoV N protein. We further investigated the shared IFN-antagonistic mechanism of MHV and SARS-CoV N proteins, and found that they targeted PACT to impede the interaction of this molecule with RIG-I/MDA5, leading to blockade of PACT-activated RIG-I/MDA5, and ultimately inhibition of IFN-β synthesis.

CoV N proteins share a similar overall topology structure and highly conserved function of packaging the viral genome with structural proteins to form ribonucleoprotein complexes for viral assembly [55]. However, their behaviors in host innate immune mediation were quite distinct from this structural function. Among CoV N proteins, MHV, SARS-CoV, and PEDV N proteins were identified as IFN antagonists [38]. MHV and SARS-CoV N proteins were proved by us to target PACT to inhibit IFN-β synthesis, but the N protein of MHV also inhibited RNaseL-mediated host translation shut-off [39], which has not yet been shown for SARS-CoV N. PEDV N protein was previously reported to target TBK1, thus disrupting the interaction between IRF3 and TBK1 [38]. Surprisingly, MERS-CoV N protein was demonstrated to be unable to suppress IFN production [56]. But recently, SARS-CoV and MERS-CoV N proteins were discrepantly reported to prohibit RIG-IN induced IFN-β production with same methods [57]. In addition, given the mutual RNA binding properties of CoV N proteins [58], it will be of interest to determine whether coating of dsRNA is another common mechanism by which N proteins antagonize IFN-β expression, whereby dsRNA is masked from immunosurveillance sensing molecules.

PACT harbors multiple functions. Among these, PACT heterodimerizes with PKR and activates it via protein–protein interactions between the dsRNA-binding domain present in each protein [59–61]. All three domains of PACT can interact with but only domain 3 activates PKR [51]. In this study, we demonstrated that domain 3 of PACT is sufficient for N proteins association, but much weakly compared with wild type PACT, which is share similarity to PACT-PKR interaction. PACT can directly interact with the CTD of RIG-I, thus stimulating its ATPase activity and mediating RIG-I activation. Here, we also identified that PACT induced MDA5 activation is via directly interaction. However, MDA5 seems to be lower activated than RIG-I upon PACT stimuli. Even though RIG-I and MDA5 share a highly homologous structure, they are activated by distinct agonists. Considering the domain of PACT which associates with and activates RIG-I/MDA5 has not been mapped, the mechanisms of PACT-mediated RIG-I/MDA5 activation and N proteins involved in PACT-RIG-I/MDA5 perturbation remain obscure. Besides, PACT associates with TAR RNA binding protein (TRBP) and/or Dicer, thus participating the RNA interference process [62–65]. In the context of different binding partners, N-PACT counteraction may involve in distinct physiological process. The Ebola virus protein VP35–PACT interaction has been verified to interfere with RIG-I activation. Furthermore, this was also proposed to contribute to functions of RNA silencing and PKR prohibition in the absence of virus infection or IFN [66–69]. Previous study suggested that SARS-CoV N protein suppresses RNA interference [70]. Whether the CoV N–PACT association can also contribute to the biogenesis of regulatory RNAs requires further study.

PACT-mediated IFN production was described by two mechanisms: PACT potentiation of the dsRNA-induced activation of RIG-I, or direct binding of PACT to the CTD of RIG-I to augment ATPase activation [24, 71]. Thereafter, an expanding list of dsRNA-binding, IFN-antagonizing, and PACT-targeting viral proteins were identified. According to the feature of PACT combination, proteins can be classified into two types: RNA dependent and RNA independent. Us11 protein of Herpes simplex virus 1, VP35 of Ebola virus, and NS1 protein of influenza A virus interactions with PACT require an RNA binding motif, and Us11 or VP35–PACT interactions are verified totally RNA independent [49, 72, 73]. This contributes to the hypothesis that Us11 and VP35 have higher affinities for PACT than RNA, to impair direct binding of PACT to RIG-I. However, targeting of PACT by ORF4a of MERS-CoV and the N proteins of MHV and SARS-CoV are mediated by RNA [48]. Mechanistically, these three proteins may compete with RIG-I/MDA5 for dsRNA and inhibit dsRNA recognition. As mentioned above, PACT can augment RNA agonists in RIG-I/MDA5 activation. However, the interaction of MHV or SARS-CoV N proteins with dsRNA and PACT is proposed to disrupt this process. Alternatively, the antagonist-RNA-PACT complex may render RNA–PACT nonfunctional in terms of RIG-I/MDA5 activation, or prevent its subsequent combination with RIG-I/MDA5.

Despite the high homology in genetic sequence [3], IFN antagonists produced by CoVs exploit divergent strategies with complicated characteristics. Although the majority of proteins from the various CoVs display the same phenotypic trends in IFN production, there are exceptions. While nsp1, -3, −7, −14, −15, and -16 from both SARS-CoV and PEDV were found to inhibit IFN production, as was PEDV nsp5, but not SARS-CoV nsp5 [33, 34, 77–82]. Thus, IFN-antagonizing activity regulated by the same antagonist with similar function from differing CoVs may do so by the same mechanism, or may not. The strategy exploited by both MERS-CoV and SARS-CoV M protein is attenuation of the formation of functional TBK1-containing complexes [83, 84]. Alternative examples include the papain-like proteases (PLPs). PLP from SARS-CoV, PEDV, and MHV can deubiquitinate innate immune modulators, but the specific targets are STING/RIG-I and STING/TBK1 [33, 85, 86]. It would appear that a strategy of tailoring several inhibitors to one target has been adopted. As demonstrated in the present study, both MHV and SARS-CoV N proteins can target PACT. There may be compensatory mechanisms involving other viral proteins to counter PACT-mediated IFN signaling in place of the N protein, such as the inhibition achieved by ORF4a protein of MERS-CoV [48]. Whether the anti-PACT mechanism could be used by other RNA-binding and IFN-antagonistic proteins requires further research, especially with regards to the proteins from the recently emerged severe pathogens MERS-CoV and PDCoV, the IFN-inhibitory effects of which have not yet been thoroughly investigated.

## MATERIALS AND METHODS

Cells, viruses, and reagents. HEK-293T and L929 cells were cultured and maintained in RPMI-1640 (HyClone, Utah, USA) and DMEM (Gibco, MA, USA), respectively, supplemented with 10% heat-inactivated fetal bovine serum (FBS) (PAN-biotech, Bavaria, Germany) at 37°C in a humidified 5% CO_2_ incubator. MHV A59 was propagated in L929 cells. Recombinant VSV-GFP was generously provided by Prof. Zhigao Bu, Harbin Veterinary Research Institute, P. R. China. SEV was obtained from the Centre of Virus Resource and Information, Wuhan Institute of Virology, Chinese Academy of Sciences. Poly(I:C), poly(I) and poly(C)-agaroses were bought from Sigma-Aldrich (MO, USA). Mouse monoclonal antibodies against Flag, HA, Myc and β-actin were purchased from Medical & Biological Laboratories (Nagoya, Japan). Rabbit polyclonal antibodies directed against Flag were bought from ABclone (Wuhan, China). Alexa Fluor 488-conjugated donkey anti-mouse and 594-conjugated donkey anti-rabbit antibodies were purchased from Santa Cruz Biotechnology (CA, United States). RNase A (Ribonuclease A) was purchased from TaKaRa Biotechnology (Dalian, China)

### Plasmids

The luciferase reporter plasmids IFN-β–Luc, 4×PRDIII/I–Luc (referred to as IRF-3–Luc), and 4×PRDII–Luc (referred to as NF-κB–Luc), and molecules in RLR signaling pathway including RIG-I, MDA5, IPS-1, TKB1, IKKε, IRF3, IRF3-5D have been described previously [38], as has the plasmid containing the N protein of PRRSV, pCAGGS–Flag-PRRSV-N [87]. pCAGGS–Flag-PEDV-N-encoded PEDV N protein was constructed with specific primers and amplified from pCAGGS–HA-PEDV-N [38]. pCMV-tag2b-SARS-CoV-N was kindly provided by Deyin Guo (Wuhan University, China). pCMV-tag2b-MHV-N was constructed by standard reverse transcription (RT)-PCR assays with specific primers to amplify the cDNA of the MHV N gene from the total RNA extracted from L929 cells infected with MHV A59. MHV, SARS-CoV, PEDV and PRRSV encoded N proteins from pCMV-tag2B or pCAGGS-Flag were constructed into pCAGGS–HA or/and pCAGGS–Myc plasmids, and were named as pCAGGS–HA-MHV-N, pCAGGS–Myc-MHV-N, pCAGGS–HA-SARS-CoV-N, pCAGGS–Myc-SARS-CoV-N, pCAGGS–Myc-PEDV-N and pCAGGS–Myc-PRRSV-N, respectively. pCAGGS–HA-PACT was similarly constructed from HEK-293T cell RNA extracts. pCAGGS–HA-ΔPACT was constructed with overlap extension PCR by using indicated primers. And all primers used in these constructs were shown in Table 1. All constructs were validated by DNA sequencing.

**Table 1:** Primers for expression plasmids construction

Primer	Sequences (5′ to 3′)
pCMV-tag2b-MHV-N-F	GAAGGATCCATGTCTTTTGTTCCTGGGCAA
pCMV-tag2b-MHV-N-R	GGGCTCGAGTTACACATTAGAGTCATCTTC
pCAGGS–HA-MHV-N-F	TAAGGTACCATGTCTTTTGTTCCTGGGCAA
pCAGGS–HA-MHV-N-R	GGGCTCGAGTTACACATTAGAGTCATCTTC
pCAGGS–Myc-SARS-CoV-N-F	GAGGAATTCATGTCTGATAATGGACCC
pCAGGS–Myc-SARS-CoV-N-R	GAGGGTACCTTATGCCTGAGTTGAATC
pCAGGS–Myc-PRRSV-N-F	GGCGAATTCATGCCAAATAACAACGGCAAGCAG
pCAGGS–Myc-PRRSV-N-R	TAAAGATCTTCATGCTGAGGGTGATGCTGTGGC
PCAGGS–Flag-PEDV-N-F	GGGGGTACCATGGCTTCTGTCAGTTTT
PCAGGS–Flag-PEDV-N-R	TTTCTCGAGTTAATTTCCTGTATCGAA
pCAGGS–HA-PACT-F	TTTGGTACCATGTCCCAGAGCAGGCACCG
pCAGGS–HA-PACT-R	GCCCTCGAGTTACTTTCTTTCTGCTATTATCT
PACT-1-34-R	ACTTGCATTGGCTTTTGTTTTCCCTGGCTTA
PACT99-126-F	GAAAACAAAAGCCAATGCAAGTAT
PACT99-126-R	TATTACTAAAATTAAGCTGGTTCTTT
PACT192-315-F	CAGCTTAATTTTAGTAATATTTCTCCAGAG

### Luciferase reporter gene assay

HEK-293T cells grown in 24-well plates were co-transfected with 0.1 μg/well reporter plasmid, 0.02 μg/well pRL–TK plasmid (Promega; as an internal control for the normalization of transfection efficiency), and the indicated expression plasmid or an empty control plasmid. Where indicated, the cells were also mock-infected/treated or infected/treated with SEV (10 hemagglutinating activity units/well)/poly(I:C) 24 h after co-transfection. The cells were lysed 12 h later and the firefly luciferase and Renilla luciferase activities were determined with the Dual-Luciferase Reporter Assay System (Promega), according to the manufacturer's protocol. The data are the relative firefly luciferase activities normalized to the Renilla luciferase activities and are representative of three independently conducted experiments. Data are presented as means and standard deviations (SD). Values of *P* < 0.05 were considered statistically significant, and *P* values of < 0.01 were considered highly statistically significant.

### RNA extraction and quantitative real-time RT-PCR

To determine the effects of MHV N protein on the expression of IFN-β, HEK-293T cells in 24-well plates were transfected with 1 μg of empty vector or plasmid encoding MHV N protein. After 24 h, the cells were mock-infected or infected with SEV for 12 h. Total RNA was extracted from the cells with RNA-Solv Reagent (Omega, GA, USA) and an aliquot (1 μg) was reverse transcribed to cDNA using AMV reverse transcriptase (Roche, Basel, Switzerland). The cDNA (1 μl of the 20 μl RT reaction) was then used as the template in a SYBR Green PCR assay (Applied Biosystems). The abundance of the individual mRNA or viral RNA transcript in each sample was assayed three times and normalized to that of the internal control, glyceraldehyde-3-phosphate dehydrogenase (GAPDH) mRNA. The primers were designed using Primer Express software v.3.0 (Applied Biosystems) as shown in Table 2.

**Table 2:** Primers used for real-time RT-PCR

Symbol	Forward primer	Reverse primer
IFN-β	tctttccatgagctacaacttgct	gcagtattcaagcctcccattc
SEV HN	aaaattacatggctaggagggaaac	gtgaatggaatggttgtgactctta
GAPDH	tcatgaccacagtccatgcc	ggatgaccttgcccacagcc

### Co-immunoprecipitation and immunoblotting analyses

For the transient transfection experiments, HEK-293T cells were transfected with the appropriate plasmids for 28 h. The transfected cells were lysed in 200 μL of lysis buffer (4% SDS, 3% DTT, 0.065 mM Tris-HCl, [pH 6.8], 30% glycerine) supplemented with protease inhibitor (PMSF, Sigma-Aldrich). The lysates were boiled at 100°C for 10 min before separation by SDS-PAGE and electroblotting onto a polyvinylidene difluoride membrane (Bio-Rad), and analyzed by immunoblotting with the indicated antibodies. For co-immunoprecipitation analysis, cells were washed with PBS and lysed for 20 min at 4°C in lysis buffer containing 50 mM Tris-HCl (pH 7.4), 150 mM NaCl, 1% NP-40, 10% glycerin, 0.1% SDS, and 2 mM Na_2_EDTA. The lysates were then cleared by centrifugation, and the proteins were immunoprecipitated overnight at 4°C with affinity antibodies and protein A+G agarose beads (Beyotime, Shanghai, China). The immunoprecipitates were washed three times with 1 mL of lysis buffer and then analyzed by standard immunoblotting procedures.

### Indirect immunofluorescence assay (IFA)

HEK-293T cells seeded on microscope coverslips and placed in 24-well dishes were co-transfected with MHV or SARS-CoV N protein expression plasmid with PACT when the cells reached approximately 70%–80% confluence. At 28 h post-transfection, the cells were fixed with 4% paraformaldehyde for 10 min, and then permeated with 0.1% Triton X-100 for 10 min at room temperature. After three washes with PBS, the cells were sealed with PBS containing 5% bovine serum albumin for 1 h, and then incubated separately with rabbit polyclonal antibody directed against Flag (1:200) or mouse monoclonal antibody directed against the HA tag (1:200) for 1 h at room temperature. The cells were then treated with Alexa Fluor 488-conjugated donkey anti-mouse or 594-conjugated donkey anti-rabbit antibodies for 1 h, and then with 4′,6-diamidino-2-phenylindole (DAPI) for 15 min at room temperature. After the samples were washed with PBS, fluorescent images were acquired with a confocal laser scanning microscope (Olympus Fluoview ver. 3.1, Tokyo, Japan).

### RNA binding assays

We assessed RNA binding ability by using poly(I:C)- or poly(C)-agarose binding assay. Poly(I:C)-coated agarose was made by incubating poly(C)-coated agarose with poly(I) in 56°C for 30 min, then cool down to 4°C. Cell lysates incubated with poly(I:C)- or poly(C)-coated agarose for 1 h in 4°C, then washed three times with lysis buffer and subjected to immunoblot analysis. Poly(C)-coated agarose was set as a negative control.

## Abbreviations

CoVs: Coronaviruses
MHV: mouse hepatitis virus
SARS: severe acute respiratory syndrome
IFN: interferon
MERS: Middle East respiratory syndrome
RIG-I: retinoic acid-induced gene I
MDA5: melanoma differentiation gene 5
PACT: interferon-induced protein kinase
PEDV: porcine epidemic diarrhea virus
PDCoV: porcine deltacoronavirus
N: nucleocapsid
VSV-GFP: vesicular stomatitis virus encoding green fluorescent protein
HA: hemagglutinin
IFA: indirect immunofluorescence assay
PRRSV: porcine reproductive and respiratory syndrome virus

## References

[1] Cavanagh D (1997). Nidovirales: a new order comprising Coronaviridae and Arteriviridae. Archives of virology.

[2] Weiss SR, Navas-Martin S (2005). Coronavirus pathogenesis and the emerging pathogen severe acute respiratory syndrome coronavirus. Microbiology and molecular biology reviews.

[3] Su S, Wong G, Shi W, Liu J, Lai AC, Zhou J, Liu W, Bi Y, Gao GF (2016). Epidemiology, Genetic Recombination, and Pathogenesis of Coronaviruses. Trends in microbiology.

[4] Woo PC, Lau SK, Lam CS, Lau CC, Tsang AK, Lau JH, Bai R, Teng JL, Tsang CC, Wang M, Zheng BJ, Chan KH, Yuen KY (2012). Discovery of seven novel Mammalian and avian coronaviruses in the genus deltacoronavirus supports bat coronaviruses as the gene source of alphacoronavirus and betacoronavirus and avian coronaviruses as the gene source of gammacoronavirus and deltacoronavirus. Journal of virology.

[5] Yang Z, Du J, Chen G, Zhao J, Yang X, Su L, Cheng G, Tang H (2014). Coronavirus MHV-A59 infects the lung and causes severe pneumonia in C57BL/6 mice. Virologica sinica.

[6] Lee N, Hui D, Wu A, Chan P, Cameron P, Joynt GM, Ahuja A, Yung MY, Leung CB, To KF, Lui SF, Szeto CC, Chung S, Sung JJ (2003). A major outbreak of severe acute respiratory syndrome in Hong Kong. The New England journal of medicine.

[7] Marra MA, Jones SJ, Astell CR, Holt RA, Brooks-Wilson A, Butterfield YS, Khattra J, Asano JK, Barber SA, Chan SY, Cloutier A, Coughlin SM, Freeman D (2003). The Genome sequence of the SARS-associated coronavirus. Science.

[8] Peiris JS, Lai ST, Poon LL, Guan Y, Yam LY, Lim W, Nicholls J, Yee WK, Yan WW, Cheung MT, Cheng VC, Chan KH, Tsang DN (2003). Coronavirus as a possible cause of severe acute respiratory syndrome. Lancet.

[9] Zaki AM, van Boheemen S, Bestebroer TM, Osterhaus AD, Fouchier RA (2012). Isolation of a novel coronavirus from a man with pneumonia in Saudi Arabia. The New England journal of medicine.

[10] Bermingham A, Chand MA, Brown CS, Aarons E, Tong C, Langrish C, Hoschler K, Brown K, Galiano M, Myers R, Pebody RG, Green HK, Boddington NL (2012). Severe respiratory illness caused by a novel coronavirus, in a patient transferred to the United Kingdom from the Middle East, September 2012. Eurosurveillance.

[11] van Boheemen S, de Graaf M, Lauber C, Bestebroer TM, Raj VS, Zaki AM, Osterhaus AD, Haagmans BL, Gorbalenya AE, Snijder EJ, Fouchier RA (2012). Genomic characterization of a newly discovered coronavirus associated with acute respiratory distress syndrome in humans. mBio.

[12] Watanabe S, Masangkay JS, Nagata N, Morikawa S, Mizutani T, Fukushi S, Alviola P, Omatsu T, Ueda N, Iha K, Taniguchi S, Fujii H, Tsuda S (2010). Bat coronaviruses and experimental infection of bats, the Philippines. Emerging infectious diseases.

[13] Yoneyama M, Fujita T (2009). RNA recognition and signal transduction by RIG-I-like receptors. Immunological reviews.

[14] Fehr AR, Perlman S (2015). Coronaviruses: an overview of their replication and pathogenesis. Methods in molecular biology.

[15] Nakhaei P, Genin P, Civas A, Hiscott J (2009). RIG-I-like receptors: sensing and responding to RNA virus infection. Seminars in immunology.

[16] Roth-Cross JK, Bender SJ, Weiss SR (2008). Murine Coronavirus Mouse Hepatitis Virus Is Recognized by MDA5 and Induces Type I Interferon in Brain Macrophages/Microglia. Journal of virology.

[17] Li JF, Liu Y, Zhang XM (2010). Murine Coronavirus Induces Type I Interferon in Oligodendrocytes through Recognition by RIG-I and MDA5. Journal of virology.

[18] Ramos HJ, Gale M (2011). RIG-I like receptors and their signaling crosstalk in the regulation of antiviral immunity. Current opinion in virology.

[19] Ireton RC, Gale M (2011). RIG-I like receptors in antiviral immunity and therapeutic applications. Viruses.

[20] Seth RB, Sun L, Ea CK, Chen ZJ (2005). Identification and characterization of MAVS, a mitochondrial antiviral signaling protein that activates NF-kappaB and IRF 3. Cell.

[21] Patel RC, Sen GC (1998). PACT, a protein activator of the interferon-induced protein kinase, PKR. The EMBO journal.

[22] Rowe TM, Sen GC (2001). Organizations and promoter analyses of the human and the mouse genes for PACT, the protein-activator of the interferon-induced protein kinase, PKR. Gene.

[23] Iwamura T, Yoneyama M, Koizumi N, Okabe Y, Namiki H, Samuel CE, Fujita T (2001). PACT, a double-stranded RNA binding protein acts as a positive regulator for type I interferon gene induced by Newcastle disease virus. Biochemical and biophysical research communications.

[24] Kok KH, Lui PY, Ng MH, Siu KL, Au SW, Jin DY (2011). The double-stranded RNA-binding protein PACT functions as a cellular activator of RIG-I to facilitate innate antiviral response. Cell host & microbe.

[25] Roth-Cross JK, Martinez-Sobrido L, Scott EP, Garcia-Sastre A, Weiss SR (2007). Inhibition of the alpha/beta interferon response by mouse hepatitis virus at multiple levels. Journal of virology.

[26] Zhou H, Perlman S (2007). Mouse hepatitis virus does not induce Beta interferon synthesis and does not inhibit its induction by double-stranded RNA. Journal of virology.

[27] Channappanavar R, Fehr AR, Vijay R, Mack M, Zhao J, Meyerholz DK, Perlman S (2016). Dysregulated Type I Interferon and Inflammatory Monocyte-Macrophage Responses Cause Lethal Pneumonia in SARS-CoV-Infected Mice. Cell host & microbe.

[28] Yen YT, Liao F, Hsiao CH, Kao CL, Chen YC, Wu-Hsieh BA (2006). Modeling the early events of severe acute respiratory syndrome coronavirus infection in vitro. Journal of virology.

[29] Hu W, Yen YT, Singh S, Kao CL, Wu-Hsieh BA (2012). SARS-CoV regulates immune function-related gene expression in human monocytic cells. Viral immunology.

[30] Cameron MJ, Kelvin AA, Leon AJ, Cameron CM, Ran L, Xu L, Chu YK, Danesh A, Fang Y, Li Q, Anderson A, Couch RC, Paquette SG (2012). Lack of innate interferon responses during SARS coronavirus infection in a vaccination and reinfection ferret model. PLoS one.

[31] Lau SK, Lau CC, Chan KH, Li CP, Chen H, Jin DY, Chan JF, Woo PC, Yuen KY (2013). Delayed induction of proinflammatory cytokines and suppression of innate antiviral response by the novel Middle East respiratory syndrome coronavirus: implications for pathogenesis and treatment. The Journal of general virology.

[32] Cao L, Ge X, Gao Y, Herrler G, Ren Y, Ren X, Li G (2015). Porcine epidemic diarrhea virus inhibits dsRNA-induced interferon-beta production in porcine intestinal epithelial cells by blockade of the RIG-I-mediated pathway. Journal of virology.

[33] Xing Y, Chen J, Tu J, Zhang B, Chen X, Shi H, Baker SC, Feng L, Chen Z (2013). The papain-like protease of porcine epidemic diarrhea virus negatively regulates type I interferon pathway by acting as a viral deubiquitinase. The journal of general virology.

[34] Zhang Q, Shi K, Yoo D (2016). Suppression of type I interferon production by porcine epidemic diarrhea virus and degradation of CREB-binding protein by nsp1. Virology.

[35] Luo J, Fang L, Dong N, Fang P, Ding Z, Wang D, Chen H, Xiao S (2016). Porcine deltacoronavirus (PDCoV) infection suppresses RIG-I-mediated interferon-beta production. Virology.

[36] Zuniga S, Cruz JL, Sola I, Mateos-Gomez PA, Palacio L, Enjuanes L (2010). Coronavirus nucleocapsid protein facilitates template switching and is required for efficient transcription. Journal of virology.

[37] Lu X, Pan J, Tao J, Guo D (2011). SARS-CoV nucleocapsid protein antagonizes IFN-beta response by targeting initial step of IFN-beta induction pathway, and its C-terminal region is critical for the antagonism. Virus Genes.

[38] Ding Z, Fang L, Jing H, Zeng S, Wang D, Liu L, Zhang H, Luo R, Chen H, Xiao S (2014). Porcine epidemic diarrhea virus nucleocapsid protein antagonizes beta interferon production by sequestering the interaction between IRF3 and TBK1. Journal of virology.

[39] Ye Y, Hauns K, Langland JO, Jacobs BL, Hogue BG (2007). Mouse hepatitis coronavirus A59 nucleocapsid protein is a type I interferon antagonist. Journal of virology.

[40] Fitzgerald KA, McWhirter SM, Faia KL, Rowe DC, Latz E, Golenbock DT, Coyle AJ, Liao SM, Maniatis T (2003). IKKepsilon and TBK1 are essential components of the IRF3 signaling pathway. Nature immunology.

[41] Sharma S, tenOever BR, Grandvaux N, Zhou GP, Lin R, Hiscott J (2003). Triggering the interferon antiviral response through an IKK-related pathway. Science.

[42] Platanias LC (2005). Mechanisms of type-I- and type-II-interferon-mediated signalling. Nature reviews Immunology.

[43] Marcus PI, Sekellick MJ (1978). Interferon action III. The rate of primary transcription of vesicular stomatitis virus is inhibited by interferon action. The Journal of general virology.

[44] Basu M, Maitra RK, Xiang Y, Meng X, Banerjee AK, Bose S (2006). Inhibition of vesicular stomatitis virus infection in epithelial cells by alpha interferon-induced soluble secreted proteins. The Journal of general virology.

[45] Sarojini S, Theofanis T, Reiss CS (2011). Interferon-induced tetherin restricts vesicular stomatitis virus release in neurons. DNA and cell biology.

[46] Yoneyama M, Kikuchi M, Natsukawa T, Shinobu N, Imaizumi T, Miyagishi M, Taira K, Akira S, Fujita T (2004). The RNA helicase RIG-I has an essential function in double-stranded RNA-induced innate antiviral responses. Nature immunology.

[47] Kato H, Takeuchi O, Sato S, Yoneyama M, Yamamoto M, Matsui K, Uematsu S, Jung A, Kawai T, Ishii KJ, Yamaguchi O, Otsu K, Tsujimura T (2006). Differential roles of MDA5 and RIG-I helicases in the recognition of RNA viruses. Nature.

[48] Siu KL, Yeung ML, Kok KH, Yuen KS, Kew C, Lui PY, Chan CP, Tse H, Woo PC, Yuen KY, Jin DY (2014). Middle east respiratory syndrome coronavirus 4a protein is a double-stranded RNA-binding protein that suppresses PACT-induced activation of RIG-I and MDA5 in the innate antiviral response. Journal of virology.

[49] Kew C, Lui PY, Chan CP, Liu X, Au SW, Mohr I, Jin DY, Kok KH (2013). Suppression of PACT-induced type I interferon production by herpes simplex virus 1 Us11 protein. Journal of virology.

[50] Huang X, Hutchins B, Patel RC (2002). The C-terminal, third conserved motif of the protein activator PACT plays an essential role in the activation of double-stranded-RNA-dependent protein kinase (PKR). The Biochemical journal.

[51] Peters GA, Hartmann R, Qin J, Sen GC (2001). Modular structure of PACT: distinct domains for binding and activating PKR. Molecular and cellular biology.

[52] Sagong M, Lee C (2011). Porcine reproductive and respiratory syndrome virus nucleocapsid protein modulates interferon-beta production by inhibiting IRF3 activation in immortalized porcine alveolar macrophages. Archives of virology.

[53] (2001). Proceedings of the VIII International Symposium on Nidoviruses (Coronaviruses and Arteriviruses). May 20–25, 2000. Lake Harmony, Pennsylvania, USA. Advances in experimental medicine and biology.

[54] Parker MM, Masters PS (1990). Sequence comparison of the N genes of five strains of the coronavirus mouse hepatitis virus suggests a three domain structure for the nucleocapsid protein. Virology.

[55] Ma Y, Tong X, Xu X, Li X, Lou Z, Rao Z (2010). Structures of the N- and C-terminal domains of MHV-A59 nucleocapsid protein corroborate a conserved RNA-protein binding mechanism in coronavirus. Protein & cell.

[56] Yang Y, Zhang L, Geng H, Deng Y, Huang B, Guo Y, Zhao Z, Tan W (2013). The structural and accessory proteins M, ORF 4a, ORF 4b, and ORF 5 of Middle East respiratory syndrome coronavirus (MERS-CoV) are potent interferon antagonists. Protein & cell.

[57] Hu Y, Li W, Gao T, Cui Y, Jin Y, Li P, Ma Q, Liu X, Cao C (2017). The Severe Acute Respiratory Syndrome Coronavirus Nucleocapsid Inhibits Type I Interferon Production by Interfering with TRIM25-Mediated RIG-I Ubiquitination. Journal of virology.

[58] McBride R, van Zyl M, Fielding BC (2014). The coronavirus nucleocapsid is a multifunctional protein. Viruses.

[59] Li S, Peters GA, Ding K, Zhang X, Qin J, Sen GC (2006). Molecular basis for PKR activation by PACT or dsRNA. Proceeding of the national academy of science of the United States of America.

[60] Singh M, Patel RC (2012). Increased interaction between PACT molecules in response to stress signals is required for PKR activation. Journal of cellular biochemistry.

[61] Daher A, Laraki G, Singh M, Melendez-Pena CE, Bannwarth S, Peters AH, Meurs EF, Braun RE, Patel RC, Gatignol A (2009). TRBP control of PACT-induced phosphorylation of protein kinase R is reversed by stress. Molecular and cellular biology.

[62] Kok KH, Ng MH, Ching YP, Jin DY (2007). Human TRBP, PACT directly interact with each other and associate with dicer to facilitate the production of small interfering RNA. The Journal of biological chemistry.

[63] Lee Y, Hur I, Park SY, Kim YK, Suh MR, Kim VN (2006). The role of PACT in the RNA silencing pathway. The EMBO journal.

[64] Noland CL, Doudna JA (2013). Multiple sensors ensure guide strand selection in human RNAi pathways. RNA.

[65] Daniels SM, Melendez-Pena CE, Scarborough RJ, Daher A, Christensen HS, El Far M, Purcell DF, Laine S, Gatignol A (2009). Characterization of the TRBP domain required for dicer interaction and function in RNA interference. BMC molecular biology.

[66] Fabozzi G, Nabel CS, Dolan MA, Sullivan NJ (2011). Ebolavirus proteins suppress the effects of small interfering RNA by direct interaction with the mammalian RNA interference pathway. Journal of virology.

[67] Feng Z, Cerveny M, Yan Z, He B (2007). The VP35 protein of Ebola virus inhibits the antiviral effect mediated by double-stranded RNA-dependent protein kinase PKR. Journal of virology.

[68] Schumann M, Gantke T, Muhlberger E (2009). Ebola virus VP35 antagonizes PKR activity through its C-terminal interferon inhibitory domain. Journal of virology.

[69] Zhu Y, Cherukuri NC, Jackel JN, Wu Z, Crary M, Buckley KJ, Bisaro DM, Parris DS (2012). Characterization of the RNA silencing suppression activity of the Ebola virus VP35 protein in plants and mammalian cells. Journal of virology.

[70] Cui L, Wang H, Ji Y, Yang J, Xu S, Huang X, Wang Z, Qin L, Tien P, Zhou X, Guo D, Chen Y (2015). The Nucleocapsid Protein of Coronaviruses Acts as a Viral Suppressor of RNA Silencing in Mammalian Cells. Journal of virology.

[71] Ho TH, Kew C, Lui PY, Chan CP, Satoh T, Akira S, Jin DY, Kok KH (2016). PACT- and RIG-I-Dependent Activation of Type I Interferon Production by a Defective Interfering RNA Derived from Measles Virus Vaccine. Journal of virology.

[72] Luthra P, Ramanan P, Mire CE, Weisend C, Tsuda Y, Yen B, Liu G, Leung DW, Geisbert TW, Ebihara H, Amarasinghe GK, Basler CF (2013). Mutual antagonism between the Ebola virus VP35 protein and the RIG-I activator PACT determines infection outcome. Cell host & microbe.

[73] Tawaratsumida K, Phan V, Hrincius ER, High AA, Webby R, Redecke V, Hacker H (2014). Quantitative proteomic analysis of the influenza A virus nonstructural proteins NS1 and NS2 during natural cell infection identifies PACT as an NS1 target protein and antiviral host factor. Journal of virology.

[74] Perlman S, Netland J (2009). Coronaviruses post-SARS: update on replication and pathogenesis. Nature reviews microbiology.

[75] Cavanagh D (2005). Coronaviruses in poultry and other birds. Avian pathology.

[76] Gralinski LE, Baric RS (2015). Molecular pathology of emerging coronavirus infections. The Journal of pathology.

[77] Narayanan K, Huang C, Lokugamage K, Kamitani W, Ikegami T, Tseng CT, Makino S (2008). Severe acute respiratory syndrome coronavirus nsp1 suppresses host gene expression, including that of type I interferon, in infected cells. Journal of virology.

[78] Wathelet MG, Orr M, Frieman MB, Baric RS (2007). Severe acute respiratory syndrome coronavirus evades antiviral signaling: role of nsp1 and rational design of an attenuated strain. Journal of virology.

[79] Huang C, Lokugamage KG, Rozovics JM, Narayanan K, Semler BL, Makino S (2011). SARS coronavirus nsp1 protein induces template-dependent endonucleolytic cleavage of mRNAs: viral mRNAs are resistant to nsp1-induced RNA cleavage. PLoS pathogens.

[80] Devaraj SG, Wang N, Chen Z, Chen Z, Tseng M, Barretto N, Lin R, Peters CJ, Tseng CT, Baker SC, Li K (2007). Regulation of IRF-3-dependent innate immunity by the papain-like protease domain of the severe acute respiratory syndrome coronavirus. The Journal of biological chemistry.

[81] Frieman M, Ratia K, Johnston RE, Mesecar AD, Baric RS (2009). Severe acute respiratory syndrome coronavirus papain-like protease ubiquitin-like domain and catalytic domain regulate antagonism of IRF3 and NF-kappaB signaling. Journal of virology.

[82] Wang D, Fang L, Shi Y, Zhang H, Gao L, Peng G, Chen H, Li K, Xiao S (2016). Porcine Epidemic Diarrhea Virus 3C-Like Protease Regulates Its Interferon Antagonism by Cleaving NEMO. Journal of virology.

[83] Siu KL, Kok KH, Ng MH, Poon VK, Yuen KY, Zheng BJ, Jin DY (2009). Severe acute respiratory syndrome coronavirus M protein inhibits type I interferon production by impeding the formation of TRAF3.TANK.TBK1/IKKepsilon complex. The Journal of biological chemistry.

[84] Lui PY, Wong LY, Fung CL, Siu KL, Yeung ML, Yuen KS, Chan CP, Woo PC, Yuen KY, Jin DY (2016). Middle East respiratory syndrome coronavirus M protein suppresses type I interferon expression through the inhibition of TBK1-dependent phosphorylation of IRF3. Emerging microbes & infections.

[85] Sun L, Xing Y, Chen X, Zheng Y, Yang Y, Nichols DB, Clementz MA, Banach BS, Li K, Baker SC, Chen Z (2012). Coronavirus papain-like proteases negatively regulate antiviral innate immune response through disruption of STING-mediated signaling. PLoS one.

[86] Wang G, Chen G, Zheng D, Cheng G, Tang H (2011). PLP2 of mouse hepatitis virus A59 (MHV-A59) targets TBK1 to negatively regulate cellular type I interferon signaling pathway. PLoS one.

[87] Song T, Fang LR, Wang D, Zhang RX, Zeng SL, An K, Chen HC, Xiao SB (2016). Quantitative interactome reveals that porcine reproductive and respiratory syndrome virus nonstructural protein 2 forms a complex with viral nucleocapsid protein and cellular vimentin. Journal of proteomics.

